# Rosiglitazone Restores Endothelial Dysfunction in a Rat Model of Metabolic Syndrome through PPARγ- and PPARδ-Dependent Phosphorylation of Akt and eNOS

**DOI:** 10.1155/2011/291656

**Published:** 2011-11-22

**Authors:** Zhigang Zhao, Zhidan Luo, Peijian Wang, Jing Sun, Hao Yu, Tingbing Cao, Yinxing Ni, Jing Chen, Zhencheng Yan, Daoyan Liu, Zhiming Zhu

**Affiliations:** Center for Hypertension and Metabolic Diseases, Department of Hypertension and Endocrinology, Daping Hospital, Third Military Medical University, Chongqing Institute of Hypertension, Chongqing 400042, China

## Abstract

Vascular endothelial dysfunction has been demonstrated in metabolic syndrome (MS). Chronic administration of rosiglitazone ameliorates endothelial dysfunction through PPAR**γ**-mediated metabolic improvements. Recently, studies suggested that single dose of rosiglitazone also has direct vascular effects, but the mechanisms remain uncertain. Here we established a diet-induced rat model of MS. The impaired vasorelaxation in MS rats was improved by incubating arteries with rosiglitazone for one hour. Importantly, this effect was blocked by either inhibition of PPAR**γ** or PPAR**δ**. In cultured endothelial cells, acute treatment with rosiglitazone increased the phosphorylation of Akt and eNOS and the production of NO. These effects were also abolished by inhibition of PPAR**γ**, PPAR**δ**, or PI3K. In conclusion, rosiglitazone improved endothelial function through both PPAR**γ**- and PPAR**δ**-mediated phosphorylation of Akt and eNOS, which might help to reconsider the complex effects and clinical applications of rosiglitazone.

## 1. Introduction

Metabolic syndrome is characterized by a combination of metabolic disorders that increase the risk of developing cardiovascular disease. Vascular endothelial dysfunction has been demonstrated in metabolic syndrome with and without hypertension or diabetes [1–3]. Thiazolidinediones (TZDs), which are a class of powerful insulin sensitizers, are ideal candidates for the early treatment of metabolic syndrome. There is also a growing body of in vivo evidence suggesting that TZDs improve endothelial function [4–7] and have a small reducing effect on blood pressure [8, 9]. The vascular protective effects might be a consequence of the metabolic changes that are mainly mediated by the transcription factor, peroxisome-proliferator-activated receptor (PPAR) *γ* [6, 8, 10, 11]. However, studies suggest that a single dose of rosiglitazone has a direct effect on endothelial function, which is independent of the metabolic improvement [12]. These studies imply that the short-term application of rosiglitazone might have endothelial benefits without any side effects, although the long-term intake of rosiglitazone is associated with an increased risk of heart failure. However, the mechanism by which rosiglitazone improves endothelial function remains uncertain. Although PPAR*γ* in endothelial cells (ECs) is involved in the regulation of nitric oxide (NO) [13], the production of reactive oxygen species [14], and the synthesis of endothelin receptor [15], rosiglitazone also has PPAR*γ*-independent effects in isolated vasculatures and cultured ECs [16].

Unlike PPAR*γ*, the most ubiquitously expressed PPAR isoform, PPAR*δ*, has been less studied in the vasculature. Previous studies have mainly focused on the metabolic role of PPAR*δ* in tissues like skeletal muscle and adipose tissue. In recent years, the role of PPAR*δ* in the vasculature has attracted more attention. PPAR*δ* in endothelial cells plays a role in the regulation of oxidative injury, inflammation, blood coagulation, cell proliferation, and apoptosis [17, 18]. One recent study revealed that PPAR*δ* agonists acutely cause vasodilatation, which is partially dependent on endothelial NO synthase (eNOS) activation through the Akt pathway [19]. PPAR*δ* activation in ECs produces an acute transcription-independent regulation of eNOS phosphorylation. Several studies indicate that rosiglitazone may enhance eNOS phosphorylation and vasorelaxation independent of genomic transcriptional regulation [16, 20]. Therefore, it is hypothesized that, in addition to PPAR*γ* activation, PPAR*δ* activation might also play an important role in the vascular protective effects of rosiglitazone.

The present study was designed to confirm the acute beneficial effects of rosiglitazone on endothelial function and to explore the mechanism by which rosiglitazone directly affects the endothelium in isolated arteries of a rat model of metabolic syndrome.

## 2. Materials and Methods

### 2.1. Animals

Male Wistar rats 6 to 8 weeks of age were obtained from an in-house breeding colony and randomized into two groups, receiving either a standard chow diet (Chow) or a high-fat diet (HFD) for 32 weeks [21]. The HFD supplied 59% of the calories as fat and 20% of the calories as carbohydrates in the form of cornstarch and sucrose (2 : 1 w/w). The Chow diet provided 10% of the calories as fat and 65% as carbohydrates. The animals were housed under controlled temperatures (21–23°C) with a 12/12 h light-dark cycle (lights on from 06:00 to 18:00). All animals had free access to water and food. Systolic blood pressure (tail-cuff method) and body weight were measured weekly. At the end of 32 weeks, rats were sacrificed after fasting for 12 h, and the second-order mesenteric arteries were used for isometric force measurement. Blood was collected from the carotid arteries, and the plasma levels of triglycerides, glucose, and insulin were measured using commercially available assay kits (Applygen Technologies Inc., Beijing, China). All of the experimental procedures were performed in accordance with protocols approved by the Institutional Animal Care and Research Advisory Committee.

### 2.2. Isometric Tension Measurement

Second-order mesenteric arteries were dissected and cut into 3 mm rings in cold Krebs solution containing the following (in mmol/L): NaCl 119, NaHCO_3_ 25, glucose 11.1, KCl 4.7, KH_2_PO_4_ 1.2, MgSO_4_ 1.2, and CaCl_2_ 2.5, with pH 7.4. Tissues were cultured for 1 h in prewarmed Dulbecco's modified Eagle medium (DMEM) supplemented with 10% Fetal Bovine Serum (FBS) and 1% antibiotics. Rosiglitazone (20 *μ*mol/L), GW9662 (PPAR*γ* antagonist, 1 *μ*mol/L), GSK0660 (PPAR*δ* antagonist, 1 *μ*mol/L), GW6471 (PPAR*α* antagonist, 1 *μ*mol/L), and the eNOS inhibitor, NG-nitro-L-arginine methyl ester (L-NAME, 100 *μ*mol/L) were added as indicated in separate experiments. Tension changes of the cultured arteries were recorded as described previously [22]. Briefly, the arterial segments were suspended by two stainless steel wires in a four-chamber wire myograph (model 610 M; Danish Myo Technology, Aarhus, Denmark) and maintained at 37°C in Krebs solution gassed with 95% O_2_ and 5% CO_2_. After a 30 min equilibration, the rings were first contracted by 10 *μ*mol/L phenylephrine (PE) and then relaxed by 1 *μ*mol/L acetylcholine (ACh). Arteries with relaxations over 80% were regarded as having an intact endothelium. After several washes, a sustained contraction was induced by adding 10 *μ*mol/L PE to the rings, and cumulative concentration relaxations using ACh (10^−9^–10^−5^ mol/L) and nitroglycerin (10^−9^–10^−5^ mol/L) were performed.

### 2.3. Cell Culture

Porcine iliac artery endothelial cells (PIECs) were obtained from the Institute of Biochemistry and Cell Biology (Chinese Academy of Sciences, Shanghai, China). PIECs were grown in DMEM supplemented with 10% FBS and 1% antibiotics. Cultures were maintained at 37°C in a humidified atmosphere of 95% air/5% CO_2_. Cells were made quiescent by incubation of 90% confluent cell cultures in serum-free DMEM and incubated with rosiglitazone (2 *μ*mol/L) for 1 h in the presence or absence of GW9662 (1 *μ*mol/L), GSK0660 (1 *μ*mol/L), GW6471 (1 *μ*mol/L), or the PI3K inhibitor, LY294002 (1 *μ*mol/L).

### 2.4. Immunoblotting

Immunoblotting of phosphorylated eNOS (Ser-1177), total eNOS, phosphorylated Akt (Ser-473), total Akt, and GAPDH in PIECs were performed using standard techniques as described previously [22, 23]. GAPDH was used as a loading control. All of the primary antibodies were purchased from Santa Cruz Biotechnology (Santa Cruz, Calif, USA). Cells were lysed in a high-salt extraction buffer (0.5 mol/L Tris, 1% NP-40, 1% Triton X-100, 1 g/L sodium dodecyl sulfate, 1.5 mol/L NaCl, 0.2 mol/L EDTA, and 0.01 mol/L EGTA) plus 0.2 mmol/L protease inhibitor and placed at −20°C for 20 minutes. Cells were centrifuged at 12,000 ×g at 4°C for 20 minutes to remove insoluble materials. Protein concentrations were determined using a DC protein assay kit (Bio-Rad, Hercules, Calif, USA). Fifty micrograms of lysates were separated by SDS-PAGE, transferred to polyvinylidene difluoride membranes and blocked with 5% nonfat milk in PBS at 37°C for 4 h. The proteins were probed with primary antibodies in 0.1% Tween-20/PBS at 4°C overnight. After incubation with the appropriate secondary antibodies at 37°C for 2 h, the proteins were detected by enhanced chemiluminescence and quantified using a Gel Doc 2000 Imager (Bio-Rad, Calif, USA).

### 2.5. Evaluation of NO Production

NO levels in PIECs were assessed using DAF-2 DA (4,5-diaminofluorescein) as described previously [22]. Cells grown on glass slides were incubated with rosiglitazone or vehicle for 1 h in fresh DMEM in the presence or absence of indicated antagonists. Then cells were washed and loaded with 2 *μ*mol/L DAF-2DA (Sigma-Aldrich) in the dark for 30 min at 37°C in Krebs solution (pH 7.4). Cells were washed three times with fresh Krebs solution. To quantitate the DAF fluorescence, the cells were observed under an inverted fluorescence microscope (Nikon TE2000, Nikon, Japan). Images were acquired and the fluorescence intensity was analyzed using NIS-Elements 3.0 software (Nikon).

### 2.6. Statistical Analysis

Data are presented as the means ± SEM. of *n* experiments. The half maximal effective agonist concentration (EC_50_) and maximum response (E_max⁡_) were calculated from individual agonist concentration-response curves using GraphPad Prism 3.0 (GraphPad Software, San Diego, Calif, USA). Statistical differences between groups were assessed using Student's *t*-test or one-way analysis of variance (ANOVA) with Bonferroni's multiple comparison post hoc tests as appropriate. Two-sided *P* values <0.05 were considered statistically significant.

## 3. Results

### 3.1. The HFD-Induced Rat Model of Metabolic Syndrome

**Figure 1:**
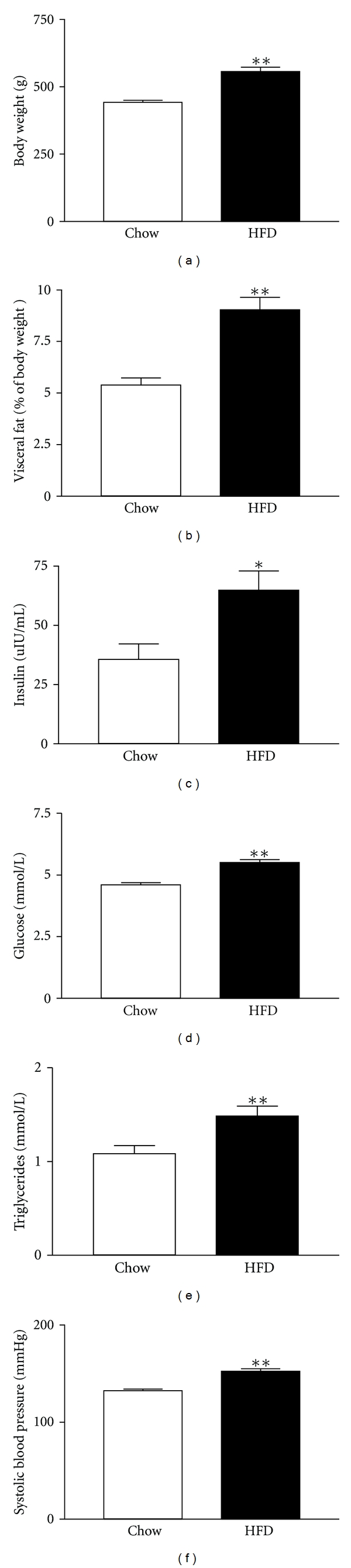
Biometric characteristics of rats on normal chow diet (Chow, *n* = 10) and high-fat diet (HFD, *n* = 12) for 32 weeks. (a) Body weight; (b) Visceral fat percentage; (c) Fasting plasma insulin level; (d) Fasting plasma glucose; (e) Fasting plasma triglycerides; (f) Systolic blood pressure by tail-cuff method. Data are means ± SEM *P < 0.05, **P < 0.01 compared to Chow.

Long-term HFD administration produced features of metabolic syndrome in Wistar-Kyoto (WKY) rats. Compared to control rats on a normal chow diet, rats on HFD had a more rapid and extreme weight gain. At the age of 32 weeks, the body weight of rats on HFD was 30% greater than control rats (Figure 1(a)). More importantly, the visceral fat component in HFD-fed rats was nearly 100% greater than controls (Figure 1(b)). As expected, HFD rats also had markedly higher fasting plasma levels of insulin, glucose, and triglycerides (Figures 1(c)–1(e)). Furthermore, the tail-cuff blood pressure of HFD-fed rats was significantly elevated compared to controls (Figure 1(f)).

### 3.2. Rosiglitazone Restored the Endothelial Dysfunction in the Rat Model of Metabolic Syndrome through Both PPAR*γ* and PPAR*δ* Activation

**Figure 2:**
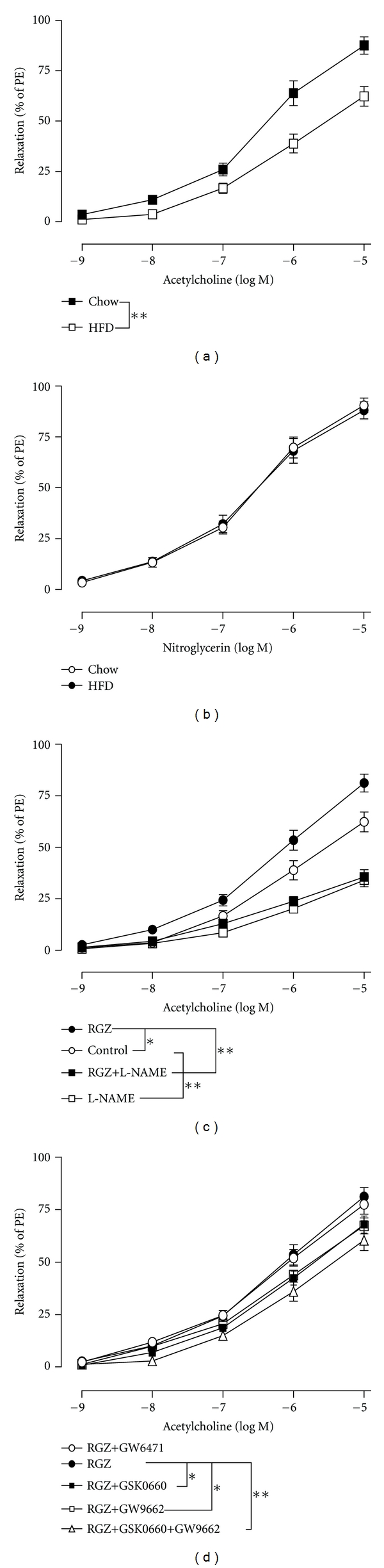
Rosiglitazone attenuated the vascular dysfunction in rats on HFD through PPAR*γ* and PPAR*δ* activation. (a, b) Acetylcholine- (a) and nitroglycerin- (b) induced relaxation in isolated mesenteric arteries from rats on normal chow diet and HFD diet. (c, d) Effects of rosiglitazone pretreatment on the Acetylcholine-induced vasorelaxation in rats on HFD in the absence or presence of L-NAME (c) or PPAR antagonists (d). RGZ: rosiglitazone; L-NAME: eNOS inhibitor; GSK0660: PPAR*δ* antagonist; GW9662: PPAR*γ* antagonist; GW6471: PPAR*α* antagonist. Data are means ± s.e.m. from 6 separate experiments. *P < 0.05, **P < 0.01 between the E_max⁡_ of indicated groups.

To exclude the multiple metabolic effects in vivo, vasorelaxation was examined in isolated mesenteric arteries of rats with or without HFD-induced metabolic syndrome. Compared to control rats, the ACh-induced endothelium-dependent vasorelaxation in HFD-fed rats was significantly impaired (Figure 2(a); HFD: E_max⁡_ = 64.94 ± 4.18%, Chow: E_max⁡_ = 89.53 ± 4.19%, *P* < 0.01), but the nitroglycerine-induced endothelium-independent vasorelaxation was similar between the two groups (Figure 2(b); HFD: E_max⁡_ = 88.01 ± 3.98%, Chow: E_max⁡_ = 91.32 ± 3.56%, *P* > 0.05). Pretreatment with rosiglitazone for 1 h markedly restored the endothelium-dependent vasorelaxation in HFD-fed rats (Figure 2(c); RGZ: E_max⁡_ = 83.66 ± 4.21%, Control: E_max⁡_ = 64.94 ± 4.18%, *P* < 0.05). In the presence of the NOS inhibitor L-NAME, the ACh-induced vasorelaxation was almost completely abolished, and there was no difference between arteries with and without rosiglitazone pretreatment (Figure 2(c)). Because rosiglitazone is a partial agonist for PPAR*γ*, we further observed an effect of rosiglitazone in the presence of antagonists for the three PPAR isoforms. The PPAR*γ* antagonist GW9662 significantly blocked the action of rosiglitazone (Figure 2(d); RGZ + GW9662: E_max⁡_ = 69.31 ± 3.10%, RGZ: E_max⁡_ = 83.66 ± 4.21%, *P* < 0.05). Surprisingly, a significant block of the action of rosiglitazone was also observed in arteries pretreated with the PPAR*δ* antagonist GSK0660 (Figure 2(d); RGZ + GSK0660: E_max⁡_ = 70.91 ± 3.45%, RGZ: E_max⁡_ = 83.66 ± 4.21%, *P* < 0.05). However, no effect of the PPAR*α* antagonist GW6471 was detected (Figure 2(d)). We further studied the vascular effects of rosiglitazone in the presence of the combination of GSK0660 and GW9662 (Figure 2(d)). It showed that the combination of GSK0660 and GW9662 had an additive inhibitory effect on vasodilation, which completely abolished the rosiglitazone-induced effect. It suggests that rosiglitazone may exert its endothelial protective role through complementary activation of PPAR*γ* and PPAR*δ*.

### 3.3. Rosiglitazone Promoted Akt and eNOS Phosphorylation through the Activation of PPAR*γ* and PPAR*δ*

**Figure 3:**
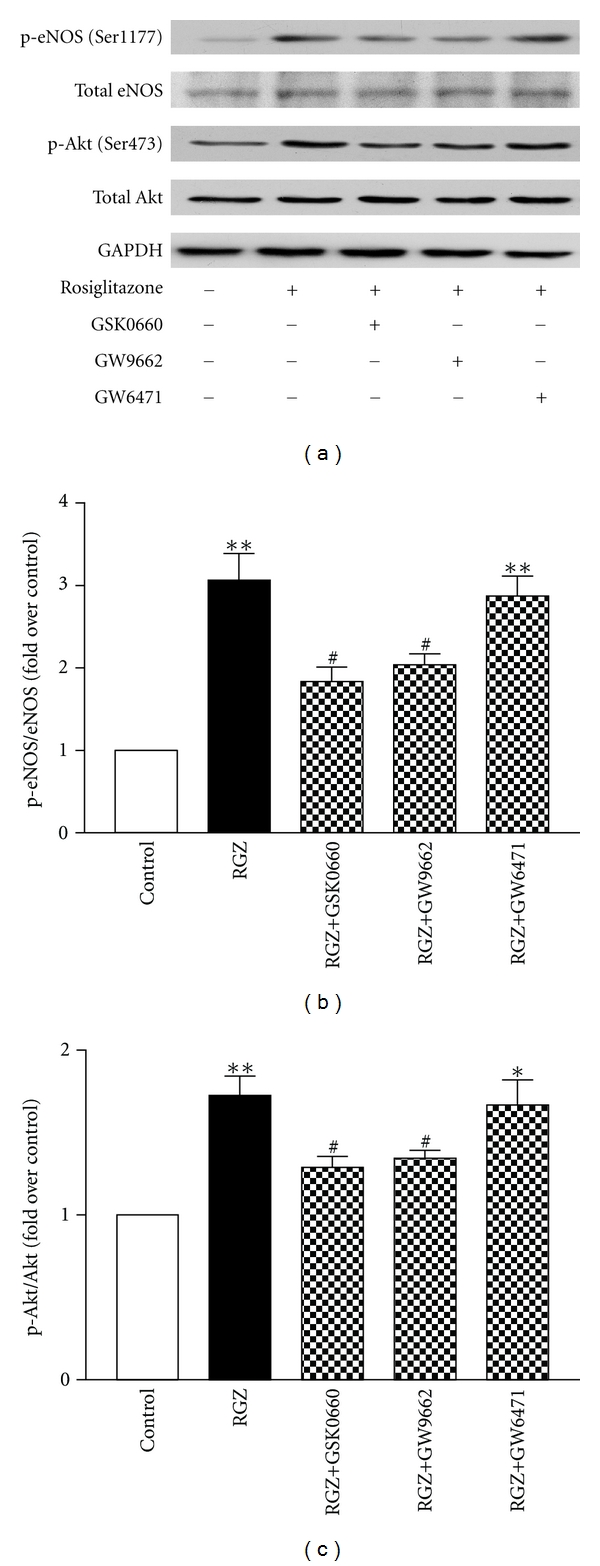
Rosiglitazone increased the phosphorylation of Akt and eNOS through PPAR*γ* and PPAR*δ* activation in cultured endothelial cells. (a) Representative immunoblots of phosphorylated and total eNOS and Akt in cells treated with rosiglitazone (RGZ) in the presence or absence of PPAR antagonists. GAPDH was used as loading control. (b, c) Summary densitometry data showing the ratio of phospho-eNOS (p-eNOS) relative to total eNOS (eNOS) and phospho-Akt (p-Akt) relative to total Akt (Akt). Data are means ± SEM from 3 separate experiments. *P < 0.05, **P < 0.01 versus Control. ^#^ *P* < 0.05 versus RGZ.

To explore the molecular mechanism involved in rosiglitazone action on the vascular endothelium, the porcine vascular endothelial cell line was cultured and treated with rosiglitazone with or without PPAR antagonists. Rosiglitazone significantly increased the phosphorylation of eNOS and Akt but had no effects on the expression of total eNOS and total Akt (Figures 3(a)–3(c)). Both antagonists for PPAR*γ* and PPAR*δ*, GW9662 and GSK0660, significantly attenuated the rosiglitazone-induced phosphorylation of Akt and eNOS (Figures 3(a)–3(c)). However, the PPAR*α* antagonist GW6471 did not affect the rosiglitazone-induced phosphorylation of eNOS or Akt (Figures 3(a)–3(c)).

### 3.4. Rosiglitazone Increased NO Production through the PPAR*γ*- and PPAR*δ*-Dependent PI3K/Akt Pathway

**Figure 4:**
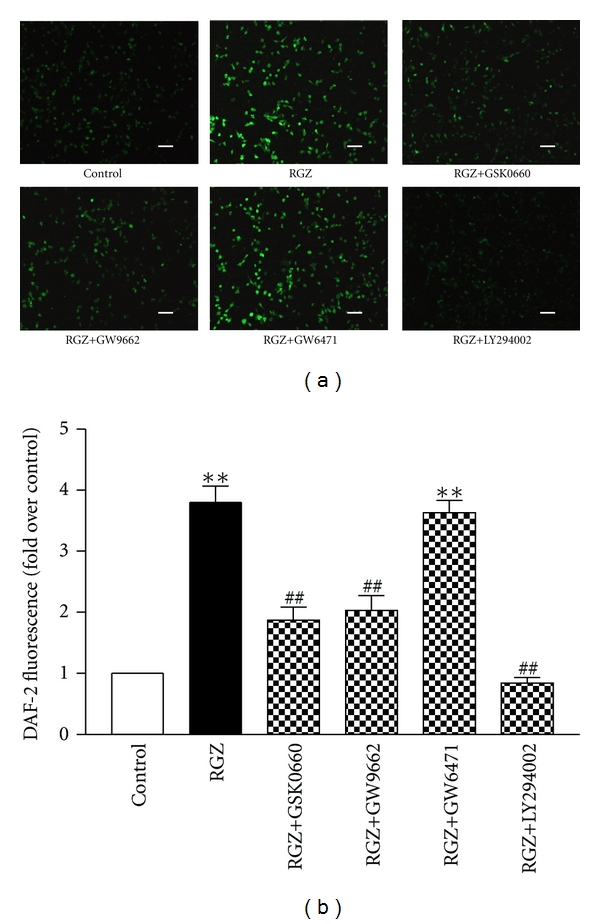
Rosiglitazone increased NO production in cultured endothelial cells through PPAR*γ*- and PPAR*δ*-dependent PI3K/Akt pathway. (a) Representative images of DAF-2 DA-loaded cells treated with rosiglitazone (RGZ) in the presence or absence of PPAR antagonists or LY294002, the PI3K inhibitor. (b) Summary data of the DAF-2 fluorescence. Data are means ± SEM from 4 separate experiments. **P < 0.01 versus Control. ^##^ *P* < 0.01 versus RGZ.

NO production in endothelial cells was determined using the DAF-2 fluorescence assay. Rosiglitazone significantly increased NO production in endothelial cells (Figures 4(a) and 4(b)). The PPAR*δ* antagonist GSK0660 and PPAR*γ* antagonist GW9662 both markedly attenuated the rosiglitazone-induced NO production (Figures 4(a) and 4(b)). However, the PPAR*α* antagonist GW6471 had no effect on the NO production in endothelial cells (Figures 4(a) and 4(b)). To determine whether the PI3K/Akt pathway was required for the rosiglitazone effect, we stimulated cells with rosiglitazone in the presence of the PI3K inhibitor LY294002 and found that rosiglitazone-induced NO production was almost completely blocked (Figures 4(a) and 4(b)).

## 4. Discussion

The present study demonstrated that rosiglitazone improved endothelial function in isolated arteries from a rat model of metabolic syndrome through both PPAR*γ*- and PPAR*δ*-mediated phosphorylation of Akt and eNOS. The short-term and direct endothelial effects of rosiglitazone and the involved new target, PPAR*δ*, might lead to a reconsideration of the complex effects and clinical applications of rosiglitazone.

Metabolic syndrome is always associated with vascular dysfunction and the common characteristic of endothelium impairment, which is the earliest known marker of atherosclerosis [1, 24, 25]. Our previous study established the diet-induced rat model of metabolic syndrome and observed multiple metabolic disorders, including central obesity, insulin resistance, dyslipidemia, and hypertension [21]. The current study further found that endothelium-dependent relaxation was impaired, but the endothelium-independent relaxation was normal in the mesenteric arteries of these rats.

A large number of in vivo studies have proven that rosiglitazone has various beneficial effects on metabolism, vascular function, and blood pressure in humans and animals [10, 11, 20]. Although the improvement of insulin sensitivity, glucose, lipids, and adiponectin could contribute to the changes in vascular function, studies in healthy humans and transgenic hypertensive animals have also shown that rosiglitazone might exhibit direct vascular effects that are independent of these metabolic actions [12, 20]. Our study confirmed the direct effects of rosiglitazone on the endothelium using short-term stimulation in isolated vessels and cultured endothelial cells.

TZDs, such as rosiglitazone, ameliorate endothelial dysfunction by stimulating NO generation through an upregulation of eNOS expression and eNOS-Ser1177 phosphorylation in animal models and cultured endothelial cells [16, 26, 27]. Our study also observed an increase in NO production and eNOS-Ser1177 phosphorylation in endothelial cells treated with rosiglitazone. However, eNOS expression was not changed by rosiglitazone. In previous studies, cells stimulated with rosiglitazone or troglitazone for up to 24 h showed changes in genomic expression [16, 26]. In the current study, cells were cultured with rosiglitazone for only 1 h. Genomic changes were not likely to occur in such a short period. Therefore, an acute non-transcriptional effect of rosiglitazone probably occurred. The phosphorylation of eNOS might involve PI3K/Akt pathway [28, 29], which could be activated by rosiglitazone [30]. In the current study, the phosphorylation of Akt and eNOS was simultaneously increased by rosiglitazone, and the PI3K inhibitor LY294002 abolished rosiglitazone-induced NO production. These results strongly support that the short-term endothelial benefits of rosiglitazone were mediated by the phosphorylation of Akt/eNOS. This acute nongenomic effect of rosiglitazone is consistent with the study of Boyle et al. who found an increase in eNOS phosphorylation and no change in total eNOS expression in cells incubated with rosiglitazone for 1-2 h [31].

Studies of the role of PPAR*γ* in the direct endothelial effects of TZDs have shown inconsistent results. In human umbilical vein endothelial cells (HUVECs), rosiglitazone-stimulated NO production and eNOS phosphorylation are completely inhibited by the PPAR*γ* antagonist GW9662 [26]. In human aortic endothelial cells, however, GW9662 has no effect on the rosiglitazone-induced acute activation of eNOS phosphorylation [31]. In the current study, the inhibition of PPAR*γ* with GW9662 partially abrogated rosiglitazone-induced NO production and Akt and eNOS phosphorylation. Although the principal function of PPAR*γ*, the known target of TZDs, is genomic regulation, it has been recently reported that PPAR*γ* physically interacts with protein kinases, which results in an acute activity modulation of these enzymes [32]. Our results support that, in addition to the chronic genomic regulation, PPAR*γ* also participates in the acute phosphorylation of Akt and eNOS.

Most importantly, the present study revealed for the first time that endothelial PPAR*δ* was activated by rosiglitazone and participated in the acute effects of rosiglitazone on endothelium-dependent vasodilation. Previous studies have found that rosiglitazone, a PPAR*γ* ligand, also shows various PPAR*γ*-independent vascular effects. Therefore, PPAR*δ* may be at least partially responsible for the PPAR*γ*-independent effects of rosiglitazone. Actually, several studies have suggested that rosiglitazone might have PPAR*δ*-dependent effects in some types of cells and tissues. Rosiglitazone inhibits lipopolysaccharide target genes in PPAR*γ*-deficient macrophages, at least in part by activating PPAR*δ* [33]. In rat synovial fibroblasts, IL-1 receptor antagonist production induced by rosiglitazone is abolished by dominant-negative PPAR*δ* and is reproduced by the PPAR*δ* agonist GW-501516 [34]. In rosiglitazone-treated obese patients with type 2 diabetes, improved insulin sensitivity and skeletal muscle oxidative enzyme activity is associated with an upregulation of PPAR*δ* expression [35]. In the current study, we revealed that rosiglitazone exhibited direct vasodilator effects partly through PPAR*δ* activation in endothelial cells. Previous studies have demonstrated that PPAR*δ* plays a direct role in various basic vascular processes, such as apoptosis, survival, angiogenesis, and inflammation [36–38]. Particularly, one recent study reported that PPAR*δ* agonists, L165041 and GW0742, acutely cause vasodilatation that is dependent on endothelial-derived NO, and the PPAR*δ*-mediated endothelial NOS activation is related to the PI3K-Akt-eNOS pathway [19]. The role of PPAR*δ* in the acute nongenomic regulation of eNOS is consistent with the findings of our current study.

In this study we only showed the vascular effects of rosiglitazone in MS rats with impaired endothelial function but not in rats with normal endothelial function. In several previous studies, rosiglitazone was shown to have vasodilator effects in normal animals and humans. Tian et al. demonstrated that rosiglitazone attenuated endothelin-1-induced vasoconstriction through the upregulation of endothelin B receptor and NO production in normal mouse aortas [15]. Hsieh and Hong reported that chronic rosiglitazone treatment augmented vascular responsiveness to acetylcholine and lowered blood pressure in normal male rats [39]. Walcher et al. showed a rapid effect of single dose rosiglitazone treatment on flow-mediated endothelium-dependent vasodilatation in healthy men [12]. These studies underscore the direct relaxation effects of rosiglitazone in normal subjects. However, it needs to clarify whether rosiglitazone can cause acute endothelium-dependent vasorelaxation through both PPAR*δ* and PPAR*γ* activation in normal animals.

In conclusion, the main finding of the present study is that, in addition to acting as a PPAR*γ* agonist, rosiglitazone also activates PPAR*δ* in endothelial cells. Both PPAR*γ* and PPAR*δ* contribute to the vasodilator effect of rosiglitazone through an increase in NO production from the phosphorylation of Akt and eNOS. The possible new target of rosiglitazone, PPAR*δ*, may promote an understanding of the wide range of properties of TZDs. More importantly, this study highlights the direct and short-term vascular effects of rosiglitazone in addition to the generally known chronic metabolic effects, and these vascular effects might provide a much safer and new clinical application of rosiglitazone.
